# High-Throughput Detection and Characterization of Antimicrobial Resistant *Enterococcus* sp. Isolates from GI Tracts of European Starlings Visiting Concentrated Animal Feeding Operations

**DOI:** 10.3390/foods9070890

**Published:** 2020-07-07

**Authors:** Jennifer Anders, Bledar Bisha

**Affiliations:** Department of Animal Science, University of Wyoming, Laramie, WY 82071, USA; andersjen121@gmail.com

**Keywords:** antimicrobial resistance, *Enterococcus* spp., high throughput detection, matrix-assisted laser desorption ionization time-of-flight mass spectrometry (MALDI-TOF MS), culture-based isolation, susceptibility testing, wildlife, environment, concentrated animal feeding operations

## Abstract

Antimicrobial resistant enteric bacteria can easily contaminate the environment and other vehicles through the deposition of human and animal feces. In turn, humans can be exposed to these antimicrobial resistant (AMR) bacteria through contaminated food products and/or contaminated drinking water. As wildlife are firmly established as reservoirs of AMR bacteria and serve as potential vectors in the constant spread of AMR, limiting contact between wildlife and livestock and effective tracking of AMR bacteria can help minimize AMR dissemination to humans through contaminated food and water. *Enterococcus* spp., which are known opportunistic pathogens, constantly found in gastrointestinal tracts of mammalian and avian species, swiftly evolve and cultivate AMR genotypes and phenotypes, which they easily distribute to other bacteria, including several major bacterial pathogens. In this study, we evaluated the use of high throughput detection and characterization of enterococci from wildlife [European starlings (*Sturnus vulgaris*)] by matrix-assisted laser desorption ionization time-of-flight mass spectrometry (MALDI-TOF MS) following culture-based isolation. MALDI-TOF MS successfully identified 658 *Enterococcus* spp. isolates out of 718 presumptive isolates collected from gastrointestinal tracts of European starlings, which were captured near livestock operations in Colorado, Iowa, Kansas, Missouri, and Texas; antimicrobial susceptibility testing was then performed using 13 clinically significant antibiotics.

## 1. Introduction

Antimicrobial resistance (AMR) is a concerning matter which currently constitutes one of the greatest threats to human and animal health [1,2]. For decades, the possibility of resistant pathogenic and commensal bacterial strains transferring from food animals and produce to humans has been a growing problem for public health, as well as a growing problem for the economy [2,3,4]. Furthermore, AMR bacteria have been continuously linked to presence in the environment and wildlife, and the possibility of resistant bacterial strains transferring to humans through the food chain is a plausible threat [4,5,6]. Because of the societal inability to keep pace with the development of antimicrobial resistance, as well as the continued use and misuse of antibiotics in the medical and agricultural fields, availability of effective antimicrobial options is dwindling [1].

All of these issues call for widespread bacterial resistance monitoring programs, which survey movements of AMR strains of bacteria to and from wildlife, livestock, and humans, monitor transfer of genetic determinants between bacteria via horizontal gene transfer, and determine current levels of resistance exhibited by bacteria [3]. The CDC has developed a four-step plan of action for the purpose of slowing the proliferation and spread of antimicrobial resistant pathogens: (1) preventing new infections and the spread of resistance; (2) detecting resistance patterns and movement; (3) improving prescription and use of antibiotics; (4) discovering new antimicrobial technologies and diagnostic testing [7]. Instead of tracking AMR pathogen movement, which can prove difficult, monitoring commensal bacteria is more convenient because of the widespread availability and ease of comparison to relevant pathogenic populations; thus, development of an “early alert system” for the transmission of microorganisms of interest to the food production system can be more easily executed [8]. Since food animals encounter wildlife, especially avian scavengers, on a continuous basis, testing of commensal bacterial samples from animals in concentrated animal feeding operations (CAFOs) and associated wildlife provides a better understanding of CAFO microbial ecology, which could be used to implement targeted programs to control the dissemination and evolution of AMR bacteria [9,10,11]. European starlings (*Sturnus vulgaris*) are commonly associated with many human and livestock pathogens and have been on the Invasive Species Specialist Group’s list “100 of the World’s Worst Invasive Alien Species” for many years, continually plaguing agricultural operations [12,13].

Areas of heavy agricultural influence are commonly associated with antimicrobial-utilizing CAFOs, dairies, and ranching operations, which are ideal habitats for synanthropic wildlife [14]. Operations such as these have recently been connected to the cultivation and harboring of new, possibly more resistant or infectious strains of pathogenic bacteria, and as such, they make an excellent source of isolates for AMR testing and tracking [15]. For this study, we evaluated matrix-assisted laser desorption ionization time-of-flight mass spectrometry (MALDI-TOF MS) for high throughput speciation of AMR enterococci found in the gastrointestinal tracts of starlings from CAFOs of Colorado, Iowa, Kansas, Missouri, and Texas. Antimicrobial susceptibility testing of 655 *Enterococcus* sp. collected from starling intestinal tracts revealed high percentages of resistant isolates to many of the 13 tested antibiotics. Such levels are rather indicative of antibiotic conditioning, and because resistance elements easily pass between commensal microorganisms and pathogens, *Enterococcus* sp. are commonly used as antimicrobial resistance indicators [16]. While MALDI-TOF MS has become an established identification method in clinical laboratories [17,18,19], the results of this study further demonstrate the utility of MALDI-TOF MS for monitoring of AMR indicators *Enterococcus* spp. in the environment and wildlife. Furthermore, this work highlights the importance of decreased contact between livestock and wildlife, continuous and meaningful antimicrobial resistance monitoring, improved reporting of antibiotic use, and the need for more judicious application of antimicrobials in agricultural settings.

Lastly, this study supported the concept that wildlife may serve as reservoirs for AMR *Enterococcus* spp. and varying levels of resistance can be found in enterococci from different geographical areas (states) as well as in different species of enterococci.

## 2. Materials and Methods 

### 2.1. Study Sites, Collection of Avian Fecal Specimens, and Culture-Based Isolation

With assistance from local and national cattlemen’s associations, National Wildlife Research Center (NWRC) staff and Wildlife Services (WS) employees, with additional help from WS offices, identified several feedlots suffering from severe starling problems. The definition of a severe starling problem for this study was any feedlot experiencing greater than 10,000 starlings per day. A total of forty sites were selected, and further information was collected for each feedlot, such as facility size, management practices, and environmental characteristics.

At each livestock facility, 30 European starlings were collected with shotguns as set forth by agency policy in the USDA/Animal and Plant Health Inspection Service (APHIS)/Wildlife Services (WS) Directive 2.505. Collection was performed by WS staff, and the facility number, time, and date of collection, and location of collection was recorded for each starling. All methods were approved by the United States Department of Agriculture (USDA) National Wildlife Research Center’s (NWRC) Animal Care and Use Committee. 

Starling carcasses were bagged individually and overnight priority shipped to the microbiology laboratory for processing.

Upon arrival at the microbiology laboratory, carcasses were rinsed with buffered peptone water (BD, Franklin Lakes, NJ, USA) to remove surface contamination. Gastrointestinal tracts from proventriculus to cloaca were removed from 1477 birds and deposited into sterile Whirl-Pak bags (Nasco, Ft. Atkinson, WI, USA). Intestines were then homogenized in a Stomacher^®^ 400 Circulator (Seward, Islandia, NY, USA) for about 120 s. Sterile, 6-inch, cotton-tipped applicators (VWR, Radnor, PA, USA) were coated with intestinal contents and used to streak compartmentalized Petri dishes (X-Plate, Fisher Scientific, Pittsburgh, PA, USA) containing Enterococcosel Agar (BD, Franklin Lakes, NJ, USA) supplemented with 4 μg/mL erythromycin (Sigma-Aldrich, St. Louis, MO, USA) (ENT-E). This approach was used to allow for the comparison of antimicrobial susceptibility profiles of *Enterococcus* spp. with important clinical resistances, such as macrolide-resistance, which also has known association to tetracycline- and glycopeptide-resistance, by limiting the subset of compared isolates to only those expected to have clinical relevance. Following inoculation, plates were incubated for 24 h at 37 °C. Colony morphology was recorded for the plates with growth, and one or two colonies were chosen for re-streaking on Enterococcosel selective media. After incubation, a single typical colony from each plate was transferred to 10 mL of brain heart infusion broth (BD, Franklin Lakes, NJ, USA), incubated for 18–24 h at 37 °C with continuous agitation, combined with 40% sterile glycerol at a 1:1 ratio, and stored at −80 °C for future use. A total of 718 presumptive *Enterococcus* spp. were collected.

### 2.2. Isolate Confirmation and Antimicrobial Susceptibility Testing

For each bacterial sample isolated using ENT-E, freezer stocks were cultured on freshly prepared brain heart infusion agar (BHIA) (BD, Franklin Lakes, NJ, USA) in the Food Microbiology Lab at the University of Wyoming. After incubation in a Heratherm Advanced Protocol Convection Oven (Thermo Scientific, Waltham, MA, USA), disposable inoculating loops (VWR, Radnor, PA, USA) were used to suspend one loopful, or about 1 μL, of bacteria in 300 μL HPLC grade water (Sigma-Aldrich, St. Louis, MO, USA), contained in a “PCR clean,” 1.5 mL Protein LoBind tube (Eppendorf, Hauppauge, NY, USA). The suspension was then vortexed with a Fisher Scientific Digital Vortex Mixer (VWR, Radnor, PA, USA), and 900 μL of absolute ethanol (Sigma-Aldrich, St. Louis, MO, USA) was added. After another thorough vortexing, the suspension was centrifuged for two minutes at 17,000× *g* using a Sorvall Legend Micro 17 Centrifuge (Thermo Scientific, Waltham, MA, USA), all ethanol was decanted, leaving a pellet containing the crude bacterial fractions. Pellets were air-dried until no ethanol remained. For further extraction of the proteins, the pellets were suspended in a 1:1 ratio of 90 μL of acetonitrile (Sigma-Aldrich, St. Louis, MO, USA) and 90 μL of 70% formic acid (Sigma-Aldrich, St. Louis, MO, USA) was added in this order; if samples were exceptionally small, a 1:1 ratio of 10 μL of acetonitrile and 10 μL of 70% formic acid was used. Vortexing was performed before and after the addition of the 70% formic acid. Once all solutions were thoroughly agitated using the vortexer, they were centrifuged again for two minutes at 17,000× *g* to remove insoluble material. After centrifugation, 1 μL of supernatant from each bacterial isolate preparation was applied to a MSP 96 polished steel BC target plate (Bruker, Billerica, MA, USA). The samples were air-dried until no liquid was visible and overlaid with 1 μL of freshly prepared α-cyano-4-hydroxycinnamic acid matrix (Bruker, Billerica, MA, USA). Once all samples were plated and overlaid, matrix-assisted laser desorption ionization, time-of-flight mass spectrometry (MALDI-TOF MS) was performed using the Bruker Ultraflex II TOF/TOF (Bruker, Billerica, MA, USA) in positive ion reflector mode. A bacterial test standard (Bruker, Billerica, MA, USA) overlaid with the matrix was used to pre-calibrate the mass spectrometer. MALDI-Biotyping was performed using the Bruker Biotyper RTC software (Version 3.1), and the highest scored identification for each sample (one spot per isolate) was recorded.

Isolates identified via MALDI-TOF MS as enterococci were chosen to undergo antibiotic disk diffusion testing using methods outlined in the Clinical and Laboratory Standards Institute’s (CLSI’s) M02-A11 protocol—*Performance Standards for Antimicrobial Disk Susceptibility Tests; Approved Standard—Eleventh Edition (2012)*—and the M100-S24 protocol—*Performance Standards for Antimicrobial Susceptibility Testing; Twenty-Fourth Informational Supplement (2014)*. In preparation for disk diffusion testing, freezer stocks of all enterococci were cultured on brain heart infusion agar, incubated for 18–24 h at 35 °C, and 3–5 colonies from each sample were chosen for the direct colony suspension method in which colonies are suspended in saline to a turbidity equal to a 0.5 McFarland standard. Mueller-Hinton Agar (MHA) (Oxoid, Basingstoke, Hampshire, UK), prepared to a depth of 4 mm, were inoculated from each of the bacterial suspensions using sterile, 6-inch, cotton-tipped applicators (VWR, Radnor, PA, USA). To ensure an even, uninterrupted lawn of growth, CLSI standards were closely followed. Thirteen antibiotics were chosen from a list of suggested antibiotics for *Enterococcus* sp. in the CLSI guidelines: rifampin (RIF), ciprofloxacin (CIP), vancomycin (VAN), erythromycin (ERY), fosfomycin (FOF), nitrofurantoin (NIT), linezolid (LZD), ampicillin (AMP), penicillin (PEN), chloramphenicol (CHL), quinupristin-dalfopristin (Q-D), doxycycline (DOX), and tetracycline (TET). Sensi-Discs (BD, Franklin Lakes, NJ, USA) impregnated with these antibiotics were applied to the inoculated agar surface using an 8-channel disk dispenser within 15 min of plate inoculation. Plates were then incubated at 35 °C for 16 to 18 h. For every isolate, the zone of inhibition around each antibiotic disk, except for vancomycin, was measured, and that measurement classified the bacterial isolate as sensitive, intermediately resistant, or resistant to the antibiotic in question. After the zones around the other antibiotic discs were measured, the plates were incubated for another 6–8 h (24 h total), and the zone of inhibition around the vancomycin disc was measured and recorded.

### 2.3. Antimicrobial Susceptibility Testing by Agar Dilution and Broth Microdilution

To further screen the isolates for vancomycin resistance, bacterial suspensions of the suspected vancomycin-resistant enterococci (VRE) and intermediately VRE were prepared and standardized to a 0.5 McFarland Standard. Ten μL of each suspension was spotted onto BHIA supplemented with 6 μg/mL vancomycin (BHIA-V) (Acumedia, Lansing, MI, USA). Plates were incubated at 35 °C for 24 h and checked for growth.

Isolates that displayed suspicious linezolid-resistant disk diffusion results and isolates that grew on BHIA-V were then tested by broth microdilution testing. Pure cultures for each isolate, grown overnight on BHIA (BD, Franklin Lakes, NJ, USA), were used to make a bacterial suspension in demineralized water, standardized to a 0.5 McFarland turbidity standard by using the Sensititre nephelometer. After vortexing, ten μL of each suspension was transferred to a manufacturer-provided tube of cation-adjusted Mueller Hinton broth (MHB), and the suspensions were vortexed again. The lids of the MHB tubes were replaced by Sensititre Dosing Heads, and the Sensititre AIM Automated Inoculation Delivery System was used to load 50 μL of each suspension into all wells of a Sensititre NARMS Gram-Positive (CMV3AGPF) plate; this plate is capable of testing a panel of antibiotics which includes tigecycline, tetracycline, chloramphenicol, daptomycin, streptomycin, tylosin tartrate, quinupristin-dalfopristin, linezolid, nitrofurantoin, penicillin, kanamycin, erythromycin, ciprofloxacin, vancomycin, lincomycin, and gentamicin. The plates were then sealed with the supplied adhesive seals, and incubated for 24 h at 36 °C. After incubation, MICs of isolates were determined and recorded for each antibiotic with the aid of a Sensititre Vizion Digital MIC Viewing System and the Sensititire Windows (SWIN) Software System (Version 3.3). All MICs were viewed and recorded by the same person and reviewed by the same person to prevent user bias. All broth microdilution supplies and instruments were obtained from Thermo Scientific (Waltham, MA, USA), unless otherwise stated.

### 2.4. Statistical Analysis by Microsoft Excel and Statistical Analysis Software

MALDI-TOF MS data and antimicrobial susceptibility data were entered into a Microsoft Excel (Version 15.33) isolate database. Using CLSI standards and Excel IFS formulas, quantitative antimicrobial susceptibility values were translated into qualitative, categorical descriptions indicating susceptible, intermediate, or resistant phenotypes. Total resistance levels for each antibiotic were calculated, as well as resistance levels for separate groups of *Enterococcus* spp., such as for *E. faecalis*, *E. hirae*, *E. faecium*, and “other” enterococci, and for enterococci from different states, using COUNTIF and COUNTIFS formulas. Numbers of multiple drug resistant isolates were calculated using COUNTIF and COUNTIFS formulas. Average numbers of antibiotics to which isolates were resistant to were calculated, using AVERAGE formulas, and averages for different groups of enterococci were compiled, using AVERAGEIF formulas. Standard deviations for all calculated means were derived by using STDEV and IF formulas. Data were also evaluated graphically. SAS was then used to make comparisons between different groups of *E. coli* and to obtain a *p*-value, describing the significance of the comparison.

A Statistical Analysis Software (SAS) program was created to analyze the Excel spreadsheets and provide measures of significance of antimicrobial susceptibility differences between groups of *Enterococcus* spp. Two-group t-tests were executed and returned *p*-values indicating the statistical significance of the differences in antimicrobial susceptibility between groups of enterococci. 

## 3. Results

### 3.1. Isolation and Confirmation of Enterococcus spp. in Starling GITs

From a total of 718 presumptive isolates obtained using subinhibitory concentrations of erythromycin added to Enterococcosel agar (ENT-E), 658 were positively identified as *Enterococcus* sp. using the Bruker Microflex LRF MALDI-TOF mass spectrometer and the Bruker Biotyper RTC software (Version 3.1). *Enterococcus faecium* was the most commonly identified species (*n* = 257), followed by *Enterococcus hirae* (*n* = 191), then *Enterococcus faecalis* (*n* = 151), *Enterococcus casseliflavus* (*n* = 32), *Enterococcus gallinarum* (*n* = 19), *Enterococcus durans* and *Enterococcus mundtii* (*n* = 3 each), and finally, *Enterococcus villorum* (*n* = 2). For this study, *E. casseliflavus*, *E. gallinarum*, *E. durans*, *E. mundtii*, and *E. villorum* were referred to as “other” enterococci (Table 1). Additional isolates identified included species from the genera *Aerococcus* (*n* = 3), *Staphylococcus* (*n* = 40), and *Vagococcus* (*n* = 3); fourteen (1.9%) of the isolates could not be reliably identified (Table 1).

For this study, genus and species identifications of isolates that returned a MALDI-TOF MS score of 1.7 or greater were accepted, although it should be noted that a score of 2.3 or above designates a “highly probable species identification,” a score between 2 and 2.299 denotes a “secure genus identification” and “probable species identification,” and a score between 1.7 and 1.999 indicates a “probable genus identification,” according to the manufacturer. Isolate-specific identification information and associated scores are presented in Table S1.

### 3.2. Antimicrobial Susceptibility Profiles

**Figure 1:**
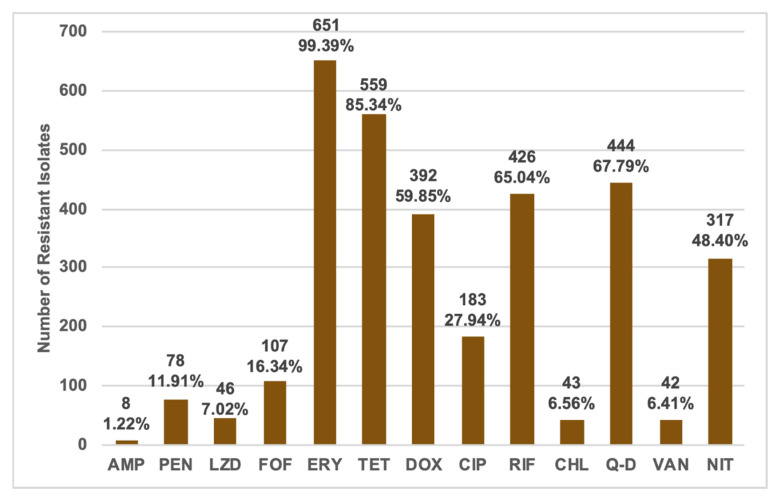
Numbers and percentages of total resistant enterococci for each of the tested antimicrobials.

As three of the identified *Enterococcus* isolates failed to show any growth on the Mueller Hinton Agar used for antimicrobial susceptibility testing after four attempts, only 655 of the *Enterococcus* sp.—255 *Enterococcus faecium* (38.93%), 190 *Enterococcus hirae* (29.01%), 151 *Enterococcus faecalis* (23.05%), 32 *Enterococcus casseliflavus* (4.89%), 19 *Enterococcus gallinarum* (2.90%), three each of *Enterococcus durans,* and *Enterococcus mundtii* (0.46% each), and two *Enterococcus villorum* (0.31%)—were characterized using the 13 clinically relevant antibiotics. Of all the isolates, 99.39% were resistant to erythromycin, 85.34% were resistant to tetracycline, 67.79% were resistant to quinupristin-dalfopristin, and 65.04% were resistant to rifampin (Figure 1).

Additionally, 59.85% of the isolates were resistant to doxycycline and 48.40% were resistant to nitrofurantoin. Few of the isolates displayed resistance to fosfomycin, chloramphenicol, and ampicillin at 16.34%, 6.56%, and 1.22%, respectively (Figure 1).

**Figure 2:**
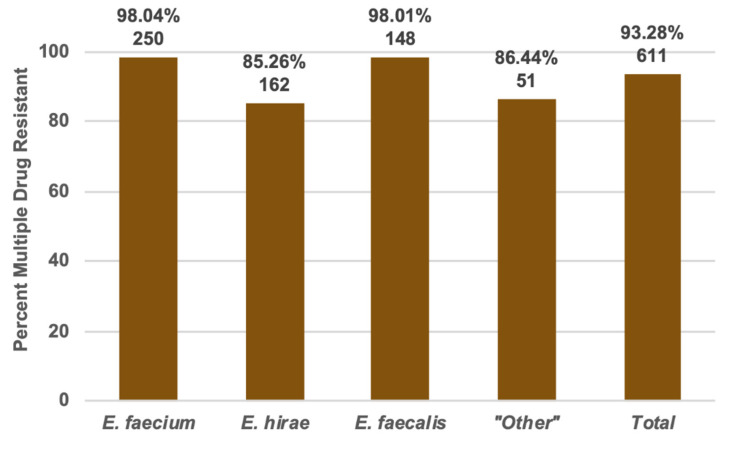
Multiple drug resistance as percent of isolates from each species and of total that were multiple drug resistant.

The 13 antibiotics used in this study comprise 11 antibiotic classes: ansamycins, fluoroquinolones, glycopeptides, macrolides, nitrofurans, oxazolidinones, penicillins, phenicols, phosphonic acids, streptogramins, and tetracyclines. Of the 655 enterococci in this study, 607 (92.67%) of them were multiple drug resistant (MDR) (Figure 2).

**Figure 3:**
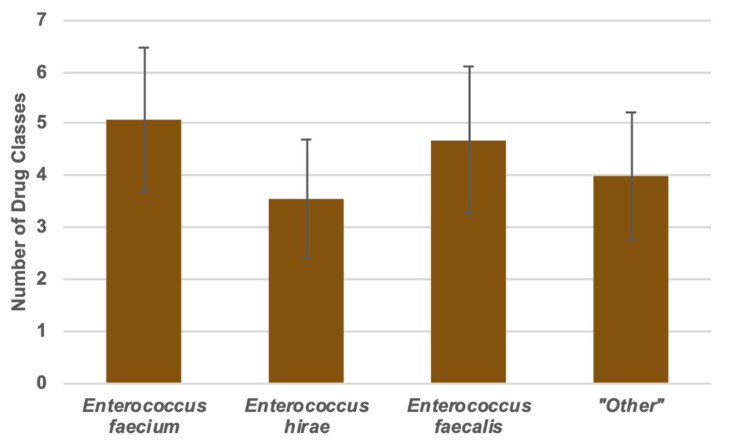
Multiple drug resistance in the form of total number of antimicrobials to which each species showed resistance (comparisons between *E. hirae* and “Other” were non-significant, *p* = 0.209).

One (0.15%) of the isolates was resistant to nine antibiotic classes, and six (0.92%) were resistant to only one antibiotic class. On average, the enterococci were resistant to 4.29 antibiotic classes. *E. faecium* isolates were resistant to 4.96 antibiotic classes, *E. hirae* isolates were resistant to 3.52 antibiotic classes, *E. faecalis* isolates were resistant to 4.34 antibiotic classes, and the “other” enterococci were resistant to 3.79 antibiotic classes, on average (Figure 3).

The only comparison that returned a non-significant *p*-value was *E. hirae* and the “other” enterococci (*p* = 0.209). The number of *E. faecalis* isolates which were MDR and the number of “other” enterococci which were MDR were significantly different (*p* < 0.001). All other grouped species comparisons returned *p*-values of less than 0.0001.

**Figure 4:**
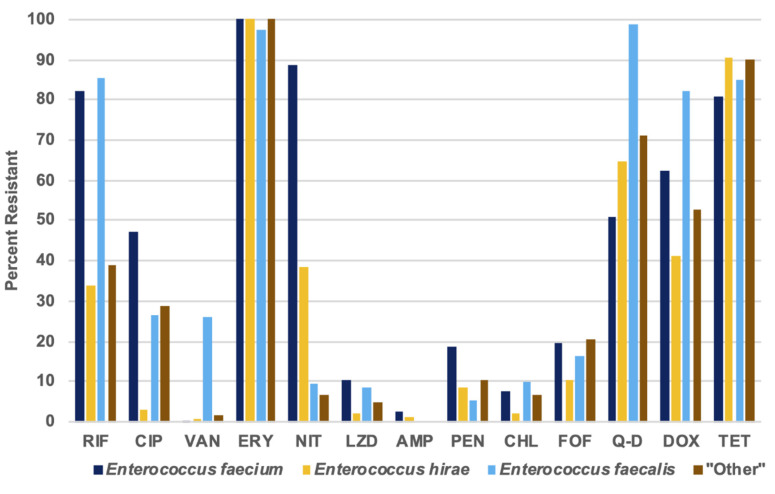
Numbers and percentages of resistance to each antimicrobial for all species of enterococci.

The *Enterococcus* isolates in this study were resistant to between one and ten of the 13 tested antibiotics; a majority of the isolates (60.00%) were resistant to five or more antibiotics and on average, the isolates were resistant to 4.90 antibiotics. Among the three *Enterococcus* spp. found with the highest frequency within this study, *E. faecium* and *E. faecalis* exhibited resistance to a noticeably higher number of antibiotics per isolate (average 5.61 and 5.16 antibiotics per isolate, respectively), than *E. hirae* isolates (average of 3.94 antibiotics per isolate). In general, greater percentages of *E. faecium* isolates displayed resistance to the tested antibiotics, except to tetracycline, doxycycline, rifampin, chloramphenicol, and quinupristin-dalfopristin. 98.68% of *E. faecalis* displayed resistance to quinupristin-dalfopristin, while only 50.98%, 64.74%, and 71.19% of *E. faecium*, *E. hirae*, and “other” enterococci displayed Q-D resistance (*p* < 0.0001 for these comparisons). More *E. faecium* isolates displayed resistance to penicillin (18.8%) than *E. hirae* isolates (8.4%, *p* < 0.001) and *E. faecalis* isolates (5.3%, *p* < 0.0001), but less *E. faecium* (10.5%) displayed resistance to fosfomycin than *E. hirae* (19.6%, *p* < 0.001). Furthermore, 90.5% of *Enterococcus hirae* isolates were resistant to tetracycline, while 80.8% (*p* < 0.05) of *E. faecium* displayed this resistance phenotype (Figure 4).

### 3.3. AMR Profile Differences between Collection Sites

**Figure 5:**
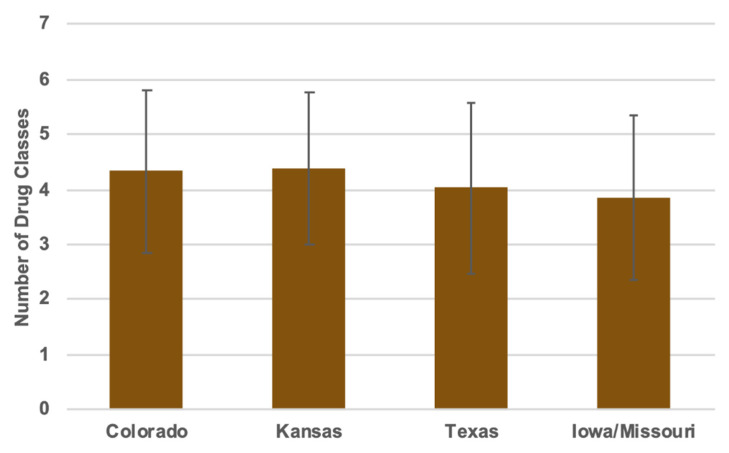
Number of antimicrobials to which isolates from each state were resistant (the only significant comparison in the panel were between Kansas and Texas, *p* = 0.019).

Of the 655 total isolates, 139 were from Colorado sites, 50 from sites in Iowa, 274 from Kansas sites, 8 from Missouri sites, and 184 were from sites in Texas. Because of the fact that the Missouri and Iowa sites produced such low numbers of isolates, the data for these states were combined into one regional group. Isolates from Kansas were resistant to an average 4.26 antibiotic classes, Colorado isolates were resistant to 4.20 antibiotic classes, Texas isolates were resistant to 3.90 antibiotic classes, and Iowa/Missouri isolates were resistant to 3.78 antibiotic classes (Figure 5).

**Figure 6:**
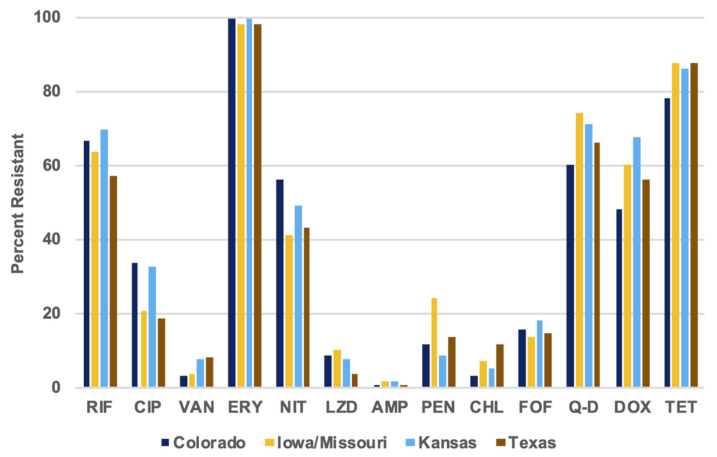
Numbers and percentages of resistance to each antimicrobial for isolates from each state.

However, 1.72%, 24.14%, and 74.14% of the Iowa/Missouri isolates displayed resistance to ampicillin, penicillin, and quinupristin-dalfopristin, while only 1.22%, 11.91%, and 67.79% of the total isolates showed those resistances. For the other antibiotics, a larger percentage of resistant isolates from Colorado, Kansas, or Texas were recorded. For tetracycline, doxycycline, chloramphenicol, and quinupristin-dalfopristin, fewer Iowa/Missouri isolates showed resistance than the isolates of other states at 78.42%, 48.2%, 2.88%, and 60.43%, respectively (Figure 6). 

All of the CAFOs from Colorado, Kansas, and Texas had at least herd sizes of 10,000 while the largest CAFO in Iowa/Missouri consisted of about 3500 head of cattle.

### 3.4. Confirmation via Broth Microdilution and Agar Dilution

Agar dilution results showed that 54 enterococci were capable of growth on BHIA-V and merited further testing.

Broth microdilution testing was used to confirm the presence of vancomycin-resistant and/or linezolid-resistant enterococci in the dataset. The isolates displaying vancomycin-resistant and intermediately vancomycin-resistant disk diffusion results were tested, using the broth microdilution technique. All of the retested isolates returned a vancomycin-susceptible result, except for one isolate. An *Enterococcus casseliflavus* isolate returned a vancomycin-resistant result. The MICs of the retested vancomycin-susceptible isolates were all between 0.5 and 8 μg/mL vancomycin, based on the manufacturer’s provided breakpoints.

## 4. Discussion

In this study, *E. faecium*, *E. hirae*, and *E. faecalis* were the most commonly isolated enterococci, in that order, reliably speciated following culture-based isolation by MALDI-TOF MS. According to an EFSA report on the guidelines for monitoring and reporting of AMR *E. coli* and *Enterococcus* spp., *E. faecium* and *E. faecalis* are the most commonly used indicator enterococci because they are routinely encountered in animal feces and most of the resistance phenotypes found in animal populations are represented by these two species [20]. According to a report generated by the European Food Safety Authority (EFSA) on AMR in zoonotic and indicator bacteria, tetracycline resistance appeared in 53.7% of the EFSA *E. faecium isolates and* 80.15% of the EFSA *E. faecalis* isolates showed resistance, while 80.78% and 84.77% of *E. faecium* and *E. faecalis* isolates in this study, respectively, showed tetracycline resistance [21]. The isolates in this study are inherently highly resistant to erythromycin because erythromycin was used in isolation. In the EFSA report, isolates from Belgium were the most resistant to erythromycin at 76.2%, but in this study, *E. faecalis* isolates showed the least percent resistance at 97.35% [21]. In Europe, 31.7% and 71.4% of *E. faecium* and *E. faecalis* isolates, respectively, were multiple drug resistant [21], but 98.04% and 98.01% of the *E. faecium* and *E. faecalis* isolates, respectively, in this study were multiple drug resistant.

Broth microdilution was used to determine antimicrobial susceptibility of *Enterococcus* spp. isolated in 2005 from the South Nation River basin in Ontario, Canada. In the study, ciprofloxacin-resistance was 2.0%, erythromycin-resistance was 0.4%, and tetracycline resistance was 1.5% [22]. In Georgia, the USDA conducted a survey of the AMR enterococci isolated from retail fruits, vegetables, and meats. Erythromycin-resistance of *E. faecalis*, *E. faecium*, and all other enterococci isolated from meats was 15%, 0%, and 1%, respectively; tetracycline resistance was 46.3%, 40% (out of 5 isolates), and 6%, respectively [23]. In a study conducted on *Enterococcus* isolates from free-living and captive raptors in Central Illinois, broth microdilution determined resistance to erythromycin and tetracycline to be 50% and 33%, respectively [24]. Other work conducted in Texas researched the antibiotic and disinfectant resistance profiles of high-level vancomycin-resistant enterococci (VRE) from community wastewater. Erythromycin- and tetracycline-resistance in these VRE isolates was 100% and 36%, respectively [25]. In a study conducted on the resistance of *Enterococcus* spp. isolated from retail meats in Iowa grocery stores, ciprofloxacin resistance of isolates from beef cuts was 19% for *E. faecium* isolates and 0% for *E. faecalis* isolates. Resistance to erythromycin in isolates from beef cuts was 8.7% for *E. faecium* and 4.5% for *E. faecalis*. Tetracycline resistance in isolates from beef cuts was 39% for both *E. faecium* and *E. faecalis* [26].

*Enterococcus* spp. can possess intrinsic resistance to certain antibiotics or can acquire antibiotic resistance through spontaneous mutations or through transfer of mobile genetic elements containing code for resistance determinants [27]. *E. faecalis* are intrinsically resistant to quinupristin and dalfopristin, B and A class streptogramins, respectively, so high levels of Q-D resistance are expected. On the other hand, *E. faecium* harbors a gene encoding an efflux pump, which confers low-level resistance to B class streptogramins only. Resistance to A class streptogramins must be acquired separately for a bacterium to be Q-D resistant [27]. Both *E. faecium* and *E. faecalis* generally possess genes which confer low-levels of resistance to β-lactams and cephalosporins via production of penicillin-binding proteins (PBPs), which bind weakly to β-lactam antibiotics [27].

Acquired enterococcal genes encoding resistance to erythromycin and tetracycline are often located on the same mobile genetic element. Transfer of these genes from enterococci isolated from food to those associated with the human gastrointestinal tract has been observed [28]. Additionally, vancomycin resistance genes generally appear on the same mobile genetic elements as the tetracycline and erythromycin resistance genes, but vancomycin resistance is less frequently encountered [28].

Iowa/Missouri isolates were resistant to fewer antibiotics, on average, compared to the isolates from Colorado, Kansas, and Texas. The CAFOs in Iowa and Missouri had much smaller cattle herds than those sampled in Colorado, Kansas, and Texas, so it is possible that smaller herd sizes result in less crowding of cattle, fewer illnesses, and therefore, lower levels of antibiotic use, which would, in turn, lower the levels of resistance displayed by the bacterial population. The differing levels of antibiotic resistance between the isolates from different states illustrates the varying practices of drug use. Contributing factors include differing drug types, drug doses, transportation of livestock, migration of wildlife, wind patterns carrying particulate matter, water runoff, crowding of animals, human antibiotic treatment, etc. [29,30].

In diagnostic testing, random and systematic errors always play a role in misclassification, but higher accuracy and minimized errors can generally be achieved with more expensive tests. In antimicrobial susceptibility testing, especially for studies with high sample numbers, the most accurate tests are not always practical or cost-effective [31]. Disk diffusion is a technique for estimating antimicrobial susceptibility of bacterial isolates, which provides results depending on the quality of growth of bacteria in the presence of varying levels of antimicrobials. Disk diffusion testing is cost-effective, and when standardized methods, established by an international body, such as the CLSI, are closely adhered to, results from disk diffusion are considered equivalent to results of other antimicrobial susceptibility testing methods [31]. Nonetheless, potential errors when using the disk diffusion method are widely recognized, and suspicious results obtained using the disk diffusion method are often corroborated or refuted by comparing to broth microdilution results, an “acceptable surrogate” for agar dilution and to agar dilution results, the “gold standard” of antimicrobial susceptibility testing [31]. Although all but two of the vancomycin-resistant and intermediately vancomycin-resistant isolates that were retested using broth microdilution returned MICs corresponding to a susceptible designation, all displayed MICs corresponding to low-levels of vancomycin resistance, suggesting partial validity of the original disk diffusion results. Agar dilution refuted all data suggesting VRE or intermediately VRE.

## 5. Conclusions

In this study, evidence was presented that MALDI-TOF MS serves as a useful tool for high throughput detection of enterococci from environmental sources (wildlife feces) following culture-based isolation. Isolation and identification of target bacteria from fecal material of wildlife is notoriously difficult, because of a number of complicating factors, including low pH, small sample size, and high non-target numbers of microorganisms; however, MALDI-TOF MS following culture-based isolation performed adequately for this purpose. Varying antimicrobial use in animals plays a part in the development of AMR strains of *Enterococcus* spp. in European starlings found in and around CAFOs. Considerable information exists outlining the selection of resistance in commensals and pathogens due to antimicrobial use in animals [32]. Since antimicrobial resistance is easily shared among bacteria, and AMR bacteria are most likely to be transferred to humans through the food chain, monitoring of resistant bacterial strains in food animals is essential in the protection of human health [32]. Because of the unavoidably important quality of antibiotics in human and veterinary medicine, steps must be taken to prevent the dissemination of AMR bacteria and genes, such as limiting wildlife contact with livestock and monitoring the use of antibiotics for any purpose.

## Figures and Tables

**Table 1:** MALDI-TOF identifications of the 718 presumptive *Enterococcus* spp. isolates initially isolated on Enterococcosel Agar supplemented with 4 μg/mL erythromycin. Of these, 658 of the isolates returned a MALDI-TOF identification of *Enterococcus* spp. For this study, genus and species identifications of isolates that returned a MALDI-TOF MS score of 1.7 or greater were accepted, although it should be noted that a score of 2.3 or above designates a “highly probable species identification,” a score between 2 and 2.299 denotes a “secure genus identification” and “probable species identification,” a score between 1.7 and 1.999 indicates a “probable genus identification,” and a score of less than 1.7 signifies no reliable identification could be determined, according to the manufacturer.

MALDI-TOF Identification	# of Isolates (%)
*Aerococcus viridans*	3 (0.4%)
*Enterococcus casseliflavus*	32 (4.5%)
*Enterococcus durans*	3 (0.4%)
*Enterococcus faecalis*	151 (21%)
*Enterococcus faecium*	257 (35.8%)
*Enterococcus gallinarum*	19 (2.6%)
*Enterococcus hirae*	191 (26.6%)
*Enterococcus mundtii*	3 (0.4%)
*Enterococcus villorum*	2 (0.3%)
*Staphylococcus aureus*	3 (0.4%)
*Staphylococcus cohnii*	9 (1.3%)
*Staphylococcus nepalensis*	6 (0.8%)
*Staphylococcus saprophyticus*	2 (0.3%)
*Staphylococcus simulans*	3 (0.4%)
*Staphylococcus xylosus*	3 (0.4%)
*Staphylococcus sciuri*	14 (1.9%)

## Supplementary Materials

The following are available online at https://www.mdpi.com/2304-8158/9/7/890/s1. Table S1: MALDI-TOF MS identification and associated scores.
