# Supplementary material for: High-Throughput Detection and Characterization of Antimicrobial Resistant Enterococcus sp. Isolates from GI Tracts of European Starlings Visiting Concentrated Animal Feeding Operations
Source: Foods. 2020 Jul 7;9(7):890. doi: 10.3390/foods9070890 (PMC7404715; doi:10.3390/foods9070890)
Supplement: Supplementary file 1 [file foods-09-00890-s001.pdf]

Table 1. MALDI-TOF MS identification and associated scores.

Isolate #	MALDI-TOF MS Identification	MALDI-TOF MS Score
1279	*Enterococcus faecalis*	2.466
1280	*Enterococcus faecium*	2.428
1281	*Enterococcus faecium*	2.452
1282	*Enterococcus faecalis*	2.407
1283	*Enterococcus faecalis*	2.531
1284	*Enterococcus faecalis*	2.494
1285	*Enterococcus faecalis*	2.442
1286	*Enterococcus faecium*	2.155
1287	*Enterococcus faecium*	2.481
1288	*Enterococcus faecium*	2.294
1289	*Enterococcus faecium*	2.212
1290	*Enterococcus faecalis*	2.201
1291	*Enterococcus faecium*	2.445
1292	*Enterococcus faecalis*	2.460
1293	*Enterococcus faecalis*	2.383
1294	*Enterococcus hirae*	2.435
1295	*Enterococcus faecalis*	2.507
1296	*Enterococcus faecalis*	2.514
1297	*Enterococcus faecalis*	2.502
1298	*Enterococcus hirae*	2.196
1299	*Enterococcus hirae*	2.395
1300	*Enterococcus hirae*	2.424
1301	*Enterococcus faecalis*	2.475
1302	*Enterococcus faecalis*	2.544
1303	*Enterococcus faecium*	2.459
1304	*Enterococcus faecium*	2.499
1305	*Enterococcus faecalis*	2.496
1306	*Enterococcus faecalis*	2.516
1307	*Enterococcus faecalis*	2.555
1308	*Enterococcus faecalis*	2.511
1309	*Enterococcus faecium*	2.559
1310	*Enterococcus faecalis*	2.543
1311	*Enterococcus faecalis*	2.491
1312	*Enterococcus faecalis*	2.307
1313	*Enterococcus faecium*	2.399
1314	*Enterococcus faecalis*	2.487
1315	*Enterococcus hirae*	2.193
1316	*Enterococcus faecalis*	2.484

Table 1. MALDI-TOF MS identification and associated scores.

1317	*Enterococcus hirae*	2.533
1318	*Enterococcus faecalis*	2.443
1319	*Enterococcus hirae*	2.546
1320	*Enterococcus faecalis*	2.500
1321	*Enterococcus faecium*	2.500
1322	*Enterococcus hirae*	2.433
1323	*Enterococcus hirae*	2.529
1324	*Enterococcus hirae*	2.380
1325	*Enterococcus hirae*	2.482
1326	*Enterococcus faecium*	2.559
1327	*Enterococcus faecalis*	2.437
1328	*Enterococcus hirae*	2.222
1329	*Enterococcus faecalis*	2.440
1330	*Staphyolcoccus simulans*	2.108
1331	*Enterococcus faecalis*	2.434
1332	*Enterococcus faecium*	2.479
1333	*Enterococcus faecalis*	2.478
1334	*Enterococcus faecalis*	2.415
1335	*Enterococcus faecium*	2.530
1336	*Enterococcus faecalis*	2.479
1337	*Enterococcus faecalis*	2.411
1339	*Enterococcus faecalis*	2.489
1340	*Enterococcus faecalis*	2.456
1341	*Enterococcus faecium*	2.387
1342	*Enterococcus faecium*	2.486
1343	*Enterococcus faecalis*	2.507
1344	*Enterococcus faecium*	2.369
1345	*Enterococcus faecium*	2.488
1346	*Enterococcus faecalis*	2.474
1347	*Enterococcus hirae*	2.360
1348	*Enterococcus faecium*	2.497
1349	*Enterococcus hirae*	2.479
1350	*Enterococcus gallinarum*	2.240
1641	*Enterococcus faecium*	2.499
1642	*Enterococcus faecium*	2.312
1643	*Enterococcus faecalis*	2.432
1644	*Enterococcus faecium*	2.542
1645	*Enterococcus faecium*	2.107
1646	*Enterococcus faecium*	2.501
1647	*Aerococcus viridans*	2.216

Table 1. MALDI-TOF MS identification and associated scores.

1648	*Enterococcus faecium*	2.443
1649	*Enterococcus hirae*	2.246
1650	*Enterococcus hirae*	2.442
1651	*Enterococcus faecalis*	2.452
1652	*Enterococcus faecium*	2.178
1653	*Enterococcus faecalis*	2.448
1654	*Staphylococcus saprophyticus*	2.128
1655	*Staphylococcus sciuri*	2.560
1656	*Enterococcus faecium*	2.329
1658	*Enterococcus hirae*	2.427
1659	*Enterococcus hirae*	2.435
1660	*Enterococcus hirae*	2.411
1661	*Enterococcus faecium*	2.489
1662	*Enterococcus faecalis*	2.485
1663	*Enterococcus faecium*	2.422
1664	*Enterococcus hirae*	2.304
1665	*Enterococcus hirae*	2.265
1666	*Enterococcus hirae*	2.408
1667	*Enterococcus hirae*	2.409
1668	*Enterococcus hirae*	2.235
1669	*Enterococcus hirae*	2.253
1670	*Enterococcus hirae*	2.249
1671	*Enterococcus faecalis*	2.414
1672	*Enterococcus faecalis*	2.408
1673	*Enterococcus faecium*	2.471
1674	*Enterococcus faecium*	2.436
1675	*Enterococcus faecium*	2.308
1676	*Enterococcus faecium*	2.521
1677	*Enterococcus hirae*	2.393
1678	*Enterococcus faecium*	2.531
1679	*Enterococcus faecium*	2.324
1680	*Enterococcus faecium*	2.475
1681	*Staphylococcus saprophyticus*	2.222
1682	*Enterococcus hirae*	2.353
1683	*Enterococcus faecium*	2.457
1684	*Enterococcus hirae*	2.370
1685	*Enterococcus faecium*	2.376
1686	*Enterococcus faecium*	2.477
1687	*Enterococcus casseliflavus*	2.362
1688	*Enterococcus faecium*	2.205

Table 1. MALDI-TOF MS identification and associated scores.

1689	*Enterococcus faecium*	2.549
1690	*Staphylococcus cohnii*	2.146
1691	*Enterococcus faecium*	2.322
1692	*Enterococcus faecalis*	2.497
1693	*Enterococcus faecium*	2.625
1694	*Enterococcus hirae*	2.448
1695	*Enterococcus faecalis*	2.516
1696	*Enterococcus faecium*	2.511
1697	*Enterococcus faecium*	2.496
1698	*Enterococcus faecalis*	2.441
1699	*Staphylococcus cohnii*	2.229
1700	*Enterococcus faecalis*	2.447
1701	*Enterococcus faecium*	2.352
1702	*Enterococcus faecium*	2.519
1703	*Enterococcus faecium*	2.617
1704	*Enterococcus faecalis*	2.502
1705	*Staphylococcus sciuri*	2.337
1706	*Enterococcus faecalis*	2.456
1707	*Enterococcus faecalis*	2.335
1708	*Enterococcus hirae*	2.456
1709	*Enterococcus faecalis*	2.594
1710	*Enterococcus faecalis*	2.549
1711	*Enterococcus faecium*	2.527
1712	*Enterococcus faecium*	2.555
1713	*Staphylococcus nepalensis*	2.228
1714	*Enterococcus faecium*	2.367
1715	*Enterococcus faecium*	2.466
1716	*Enterococcus faecalis*	2.472
1717	*Enterococcus faecium*	2.415
1718	*Enterococcus faecium*	2.392
1719	*Enterococcus faecium*	2.499
1720	*Enterococcus faecium*	2.546
1721	*Enterococcus faecium*	2.480
1722	*Enterococcus faecium*	2.296
1723	*Enterococcus hirae*	2.470
1724	*Enterococcus faecalis*	2.461
1725	*Enterococcus faecalis*	2.500
1726	*Enterococcus hirae*	2.456
1727	*Enterococcus faecalis*	2.522
1728	*Enterococcus faecium*	2.586

Table 1. MALDI-TOF MS identification and associated scores.

1729	*Enterococcus faecalis*	2.565
1730	*Enterococcus faecium*	2.572
1731	*Enterococcus faecium*	2.531
1732	*Enterococcus faecalis*	2.484
1733	*Enterococcus faecium*	2.356
1734	*Enterococcus gallinarum*	2.340
1735	*Enterococcus faecalis*	2.514
1736	*Enterococcus faecium*	2.583
1737	*Enterococcus faecium*	2.580
1738	*Enterococcus faecalis*	2.518
1739	*Enterococcus faecium*	2.604
1740	*Enterococcus faecalis*	2.537
1741	*Enterococcus faecium*	2.617
1742	*Enterococcus faecalis*	2.459
1743	*Enterococcus faecium*	2.305
1744	*Enterococcus faecalis*	2.552
1745	*Enterococcus faecium*	2.277
1746	*Enterococcus hirae*	2.471
1747	*Enterococcus faecium*	2.526
1748	*Enterococcus hirae*	2.486
1749	*Enterococcus faecium*	2.482
1750	*Enterococcus faecalis*	2.541
1751	*Enterococcus faecium*	2.590
1752	*Enterococcus hirae*	2.398
1753	*Enterococcus faecium*	2.574
1754	*Staphylococcus xylosus*	1.819
2031	*Enterococcus faecalis*	2.510
2032	*Staphylococcus simulans*	2.221
2033	*Enterococcus hirae*	2.503
2034	*Enterococcus faecium*	2.542
2035	*Aerococcus viridans*	2.074
2036	*Enterococcus faecalis*	2.483
2037	*Enterococcus hirae*	2.511
2038	*Enterococcus faecium*	2.570
2039	*Enterococcus faecalis*	2.424
2040	*Enterococcus faecalis*	2.448
2041	*Enterococcus faecium*	2.593
2042	*Enterococcus faecalis*	2.468
2043	*Enterococcus faecium*	2.422
2044	*Enterococcus hirae*	2.392

Table 1. MALDI-TOF MS identification and associated scores.

2045	*Staphylococcus cohnii*	2.259
2046	*Enterococcus faecium*	2.487
2047	*Enterococcus hirae*	2.466
2048	*Enterococcus faecium*	2.574
2049	*Enterococcus faecalis*	2.514
2050	*Enterococcus faecalis*	2.511
2051	*Enterococcus faecium*	2.567
2052	*Enterococcus hirae*	2.407
2053	*Enterococcus faecium*	2.162
2054	*Enterococcus faecalis*	2.523
2055	*Enterococcus faecalis*	2.509
2056	*Enterococcus faecalis*	2.491
2057	*Enterococcus hirae*	2.479
2058	*Enterococcus casseliflavus*	2.293
2059	*Enterococcus faecium*	2.564
2060	*Enterococcus hirae*	2.490
2061	*Enterococcus gallinarum*	2.307
2063	*Enterococcus gallinarum*	2.323
2064	*Enterococcus faecium*	2.243
2067	*Enterococcus faecium*	2.576
2068	*Staphylococcus nepalensis*	2.185
2069	*Enterococcus faecium*	2.635
2070	*Enterococcus hirae*	2.440
2071	*Enterococcus hirae*	2.474
2072	*Enterococcus faecium*	2.374
2073	*Enterococcus faecium*	2.506
2074	*Enterococcus faecium*	2.574
2075	*Enterococcus hirae*	2.304
2076	*Enterococcus hirae*	2.440
2077	*Enterococcus faecalis*	2.459
2078	NO RELIABLE ID	1.595
2079	*Enterococcus faecalis*	2.505
2080	*Enterococcus hirae*	2.500
2081	*Enterococcus hirae*	2.438
2082	*Enterococcus faecium*	2.589
2083	*Enterococcus faecalis*	2.365
2084	*Enterococcus faecalis*	2.376
2085	*Staphylococcus simulans*	2.202
2086	*Enterococcus hirae*	2.431
2087	*Enterococcus faecalis*	2.403

Table 1. MALDI-TOF MS identification and associated scores.

2088	*Enterococcus hirae*	2.419
2089	*Staphylococcus cohnii*	2.193
2090	*Enterococcus faecium*	2.604
2091	*Enterococcus faecium*	2.581
2092	NO RELIABLE ID	1.639
2093	*Enterococcus faecalis*	2.474
2094	*Enterococcus faecium*	2.566
2095	*Enterococcus faecium*	2.530
2096	*Enterococcus hirae*	2.509
2097	*Enterococcus faecalis*	2.391
2098	*Enterococcus faecalis*	2.430
2099	*Enterococcus faecium*	2.584
2100	*Enterococcus faecium*	2.404
2101	*Enterococcus faecalis*	2.469
2102	*Enterococcus hirae*	2.484
2103	*Enterococcus faecium*	2.573
2104	*Enterococcus faecalis*	2.463
2105	*Enterococcus hirae*	2.423
2106	*Enterococcus faecium*	2.480
2107	*Enterococcus faecalis*	2.418
2108	*Enterococcus faecium*	2.334
2109	*Enterococcus faecium*	2.467
2110	*Enterococcus hirae*	2.454
2111	*Enterococcus hirae*	2.456
2112	*Enterococcus faecalis*	2.521
2113	*Enterococcus faecalis*	2.463
2217	*Enterococcus faecium*	2.439
2218	*Staphylococcus xylosus*	1.705
2219	*Staphylococcus xylosus*	1.713
2220	*Enterococcus faecalis*	2.512
2221	*Staphylococcus cohnii*	2.104
2222	*Staphylococcus cohnii*	2.059
2223	*Enterococcus hirae*	2.455
2224	*Enterococcus faecium*	2.611
2225	*Enterococcus durans*	2.226
2226	*Enterococcus hirae*	2.432
2267	*Enterococcus casseliflavus*	2.441
2268	*Enterococcus faecalis*	2.486
2269	*Enterococcus faecium*	2.281
2270	*Enterococcus hirae*	2.481

Table 1. MALDI-TOF MS identification and associated scores.

2271	*Enterococcus hirae*	2.441
2272	*Enterococcus faecium*	2.285
2273	*Enterococcus hirae*	2.435
2274	*Enterococcus faecium*	2.355
2275	NO RELIABLE ID	1.595
2276	*Enterococcus faecium*	2.404
2277	*Enterococcus hirae*	2.509
2278	*Enterococcus faecium*	2.360
2279	*Enterococcus hirae*	2.435
2280	*Enterococcus hirae*	2.492
2281	*Enterococcus faecium*	2.307
2282	*Enterococcus faecium*	2.372
2283	*Enterococcus faecalis*	2.566
2284	*Enterococcus hirae*	2.445
2285	*Enterococcus faecium*	2.425
2286	*Enterococcus hirae*	2.460
2292	*Enterococcus casseliflavus*	2.236
2293	*Enterococcus villorum*	2.494
2294	*Enterococcus casseliflavus*	2.290
2295	*Enterococcus hirae*	2.417
2296	*Enterococcus casseliflavus*	2.379
2297	*Enterococcus casseliflavus*	2.447
2298	*Enterococcus faecium*	2.600
2299	*Enterococcus casseliflavus*	2.411
2301	*Enterococcus hirae*	2.429
2302	*Enterococcus gallinarum*	2.312
2303	*Enterococcus casseliflavus*	2.452
2304	*Enterococcus hirae*	2.530
2305	*Enterococcus hirae*	2.364
2306	*Enterococcus casseliflavus*	2.273
2307	*Enterococcus casseliflavus*	2.399
2308	*Enterococcus casseliflavus*	2.396
2315	*Enterococcus faecium*	2.449
2316	*Enterococcus hirae*	2.388
2317	*Enterococcus hirae*	2.427
2318	*Enterococcus hirae*	2.479
2319	*Enterococcus hirae*	2.376
2320	*Enterococcus faecium*	2.562
2321	*Enterococcus hirae*	2.446
2322	*Enterococcus hirae*	2.482

Table 1. MALDI-TOF MS identification and associated scores.

2323	*Enterococcus faecium*	2.548
2324	*Enterococcus faecalis*	2.508
2464	*Staphylococcus sciuri*	2.238
2465	*Enterococcus hirae*	2.462
2466	*Staphylococcus sciuri*	2.191
2467	*Enterococcus faecium*	2.344
2468	*Enterococcus faecium*	2.289
2469	*Enterococcus faecium*	2.291
2470	*Staphylococcus sciuri*	2.239
2471	*Staphylococcus sciuri*	2.302
2472	*Enterococcus faecium*	2.377
2473	*Enterococcus casseliflavus*	2.293
2474	*Enterococcus faecium*	2.290
2475	*Enterococcus faecium*	2.330
2505	*Enterococcus faecium*	2.300
2506	*Enterococcus faecium*	2.379
2507	*Enterococcus faecium*	2.195
2509	*Enterococcus faecalis*	2.465
2510	*Enterococcus faecalis*	2.400
2511	*Enterococcus hirae*	2.445
2513	*Enterococcus hirae*	2.314
2560	*Staphylococcus cohnii*	1.792
2561	*Enterococcus hirae*	2.391
2562	*Enterococcus hirae*	2.339
2563	*Staphylococcus sciuri*	2.258
2564	NO RELIABLE ID	1.639
2565	NO RELIABLE ID	1.413
2566	*Staphylococcus cohnii*	1.811
2567	*Staphylococcus sciuri*	2.129
2568	*Enterococcus faecium*	2.602
2569	*Enterococcus casseliflavus*	2.418
2570	*Staphylococcus nepalensis*	2.323
2571	*Enterococcus hirae*	2.492
2572	*Enterococcus hirae*	2.439
2573	*Enterococcus faecium*	2.540
2574	*Enterococcus hirae*	2.445
2575	*Enterococcus hirae*	2.476
2576	*Enterococcus hirae*	2.383
2577	*Enterococcus hirae*	2.427
2578	*Enterococcus faecium*	2.550

Table 1. MALDI-TOF MS identification and associated scores.

2579	*Enterococcus hirae*	2.364
2580	*Staphylococcus nepalensis*	2.166
2582	*Aerococcus viridans*	2.018
2620	*Enterococcus mundtii*	2.127
2621	*Enterococcus faecium*	2.553
2622	*Enterococcus faecium*	2.377
2623	*Enterococcus faecium*	2.560
2624	*Enterococcus faecium*	2.334
2625	*Enterococcus faecium*	2.321
2626	*Enterococcus faecium*	2.276
2627	*Enterococcus hirae*	2.430
2628	*Enterococcus faecalis*	2.377
2629	*Enterococcus faecalis*	2.391
2630	*Enterococcus faecium*	2.474
2631	*Enterococcus mundtii*	2.251
2632	*Enterococcus faecium*	2.577
2633	*Enterococcus faecium*	2.454
2634	*Enterococcus durans*	2.187
2635	*Enterococcus faecium*	2.527
2636	*Enterococcus faecium*	2.527
2649	*Vagococcus lutrae*	2.049
2650	*Enterococcus faecalis*	2.433
2651	*Staphylococcus sciuri*	1.866
2652	*Enterococcus casseliflavus*	2.179
2653	*Vagococcus lutrae*	2.185
2654	*Vagococcus lutrae*	1.932
2740	*Enterococcus hirae*	2.257
2741	*Enterococcus faecium*	2.286
2742	*Enterococcus casseliflavus*	2.409
2743	*Enterococcus faecium*	2.526
2744	*Staphylococcus cohnii*	1.708
2745	*Enterococcus faecalis*	2.431
2746	*Enterococcus faecium*	2.256
2747	*Enterococcus faecium*	2.344
2748	*Enterococcus gallinarum*	2.308
2749	NO RELIABLE ID	1.611
2750	*Enterococcus faecium*	2.357
2751	*Enterococcus faecium*	2.592
2752	*Enterococcus faecium*	2.346
2753	*Enterococcus faecium*	2.402

Table 1. MALDI-TOF MS identification and associated scores.

2754	*Enterococcus faecium*	2.369
2755	*Enterococcus hirae*	2.414
2756	NO RELIABLE ID	1.668
2797	*Enterococcus hirae*	2.470
2798	*Enterococcus faecalis*	2.513
2799	*Enterococcus faecalis*	2.515
2800	*Enterococcus gallinarum*	2.246
2801	*Enterococcus faecalis*	2.493
2802	*Enterococcus faecalis*	2.514
2803	*Enterococcus faecalis*	2.461
2804	*Enterococcus faecium*	2.554
2805	*Enterococcus faecium*	2.577
2806	*Enterococcus faecium*	2.313
2807	*Enterococcus faecium*	2.386
2808	*Enterococcus faecalis*	2.513
2809	*Enterococcus hirae*	2.455
2810	*Enterococcus faecalis*	2.530
2811	*Enterococcus faecium*	2.525
2812	*Enterococcus faecalis*	2.519
2813	*Enterococcus faecium*	2.585
2814	*Enterococcus faecium*	2.416
2815	*Enterococcus hirae*	2.464
2816	*Enterococcus faecium*	2.380
2817	*Enterococcus faecium*	2.535
2818	*Enterococcus faecalis*	2.482
2819	*Enterococcus faecium*	2.512
2820	*Enterococcus hirae*	2.506
2821	*Enterococcus faecium*	2.284
2822	*Enterococcus hirae*	2.496
2823	*Enterococcus faecium*	2.532
2824	*Enterococcus faecium*	2.477
2825	*Enterococcus faecium*	2.592
2826	*Enterococcus faecium*	2.401
2827	*Enterococcus faecalis*	2.460
2828	*Enterococcus faecium*	2.419
2829	*Enterococcus faecium*	2.417
2830	*Enterococcus faecium*	2.585
2831	*Enterococcus casseliflavus*	2.389
2832	*Enterococcus faecium*	2.452
2833	*Enterococcus faecalis*	2.465

Table 1. MALDI-TOF MS identification and associated scores.

2834	*Enterococcus hirae*	2.387
2835	*Enterococcus hirae*	2.418
2836	*Enterococcus faecalis*	2.542
2837	*Enterococcus faecalis*	2.521
2838	*Enterococcus faecium*	2.500
2881	*Enterococcus faecium*	2.406
2882	*Enterococcus gallinarum*	2.384
2883	*Enterococcus hirae*	2.515
2884	*Enterococcus hirae*	2.379
2885	*Staphylococcus aureus*	2.439
2886	*Enterococcus hirae*	2.440
2887	*Enterococcus faecium*	2.199
2888	*Enterococcus hirae*	2.430
2889	*Enterococcus hirae*	2.350
2890	*Enterococcus hirae*	1.945
2891	*Enterococcus faecium*	2.326
2892	*Enterococcus hirae*	2.396
2893	*Enterococcus hirae*	2.410
2944	*Enterococcus hirae*	2.199
2945	*Enterococcus hirae*	2.439
2946	*Enterococcus hirae*	2.244
2947	*Enterococcus faecium*	2.248
2949	*Enterococcus hirae*	2.401
2950	*Staphylococcus nepalensis*	1.884
2951	*Enterococcus faecium*	2.321
2952	*Enterococcus villorum*	2.375
2975	*Enterococcus hirae*	2.375
2976	*Enterococcus hirae*	2.369
2977	*Enterococcus hirae*	2.439
2978	*Enterococcus hirae*	2.279
2979	*Enterococcus casseliflavus*	2.154
2980	*Enterococcus durans*	1.993
2981	*Enterococcus faecium*	2.239
2982	*Enterococcus hirae*	2.395
2983	*Enterococcus hirae*	2.472
2984	*Enterococcus faecium*	2.572
2985	*Enterococcus faecalis*	2.460
2986	*Enterococcus faecalis*	2.340
2987	*Enterococcus hirae*	2.361
2988	*Enterococcus faecalis*	2.519

Table 1. MALDI-TOF MS identification and associated scores.

2989	*Enterococcus hirae*	2.387
2990	*Enterococcus hirae*	2.471
2991	*Enterococcus gallinarum*	2.361
2992	*Enterococcus hirae*	2.360
2993	*Enterococcus hirae*	2.390
2994	*Enterococcus faecium*	2.207
3090	*Enterococcus faecalis*	2.506
3091	*Enterococcus hirae*	2.339
3092	*Enterococcus hirae*	2.341
3093	*Enterococcus faecium*	2.428
3094	*Enterococcus hirae*	2.459
3095	*Enterococcus hirae*	2.448
3096	*Enterococcus hirae*	2.418
3097	*Enterococcus hirae*	2.309
3098	*Enterococcus faecium*	2.404
3099	*Enterococcus faecium*	2.092
3100	*Staphylococcus nepalensis*	1.832
3101	NO RELIABLE ID	1.615
3102	*Enterococcus casseliflavus*	2.200
3103	*Enterococcus hirae*	2.451
3104	*Enterococcus hirae*	2.489
3105	*Enterococcus faecium*	2.272
3106	*Enterococcus hirae*	2.500
3169	*Enterococcus faecium*	2.357
3170	*Enterococcus casseliflavus*	2.149
3171	*Enterococcus hirae*	2.459
3172	*Enterococcus casseliflavus*	2.340
3173	*Enterococcus hirae*	2.340
3174	*Enterococcus faecium*	2.331
3175	*Enterococcus hirae*	2.430
3176	*Enterococcus casseliflavus*	2.227
3177	*Enterococcus hirae*	2.368
3178	*Enterococcus hirae*	2.382
3179	*Enterococcus faecium*	2.266
3180	*Enterococcus hirae*	2.370
3181	*Enterococcus faecium*	2.200
3182	*Enterococcus faecalis*	2.528
3183	*Enterococcus faecalis*	2.251
3184	*Enterococcus faecalis*	2.327
3185	*Enterococcus faecium*	2.345

Table 1. MALDI-TOF MS identification and associated scores.

3186	*Enterococcus faecalis*	2.333
3187	*Enterococcus faecalis*	2.323
3188	*Enterococcus casseliflavus*	2.220
3189	*Enterococcus faecium*	2.391
3190	*Enterococcus faecalis*	2.329
3191	*Enterococcus faecium*	2.439
3192	*Enterococcus faecium*	2.174
3193	*Enterococcus faecalis*	2.305
3194	*Enterococcus faecium*	2.323
3195	*Enterococcus faecalis*	2.306
3196	*Enterococcus casseliflavus*	2.156
3197	*Enterococcus faecium*	2.463
3198	*Enterococcus hirae*	2.322
3199	*Enterococcus casseliflavus*	2.244
3200	NO RELIABLE ID	1.515
3201	*Enterococcus faecium*	2.209
3202	*Enterococcus faecalis*	2.299
3203	*Enterococcus faecalis*	2.189
3204	*Enterococcus faecium*	2.205
3205	*Enterococcus faecalis*	2.263
3206	*Enterococcus faecium*	2.490
3207	*Enterococcus faecium*	1.906
3208	*Enterococcus faecium*	2.352
3209	*Enterococcus faecium*	2.329
3210	*Enterococcus faecium*	2.309
3211	*Enterococcus faecium*	2.445
3212	*Enterococcus faecalis*	2.235
3213	*Enterococcus faecium*	2.387
3214	NO RELIABLE ID	1.452
3215	*Enterococcus gallinarum*	2.096
3216	*Enterococcus gallinarum*	1.922
3217	*Enterococcus hirae*	2.180
3218	*Enterococcus faecium*	2.365
3219	*Enterococcus gallinarum*	2.008
3220	*Enterococcus faecalis*	2.202
3221	*Enterococcus faecalis*	2.282
3222	*Enterococcus faecium*	2.337
3223	*Enterococcus faecium*	2.447
3224	*Enterococcus faecium*	2.371
3225	*Enterococcus gallinarum*	2.045

Table 1. MALDI-TOF MS identification and associated scores.

3226	NO RELIABLE ID	1.485
3227	NO RELIABLE ID	1.646
3228	*Enterococcus casseliflavus*	2.193
3229	*Enterococcus faecalis*	2.274
3230	*Enterococcus faecalis*	2.120
3231	*Enterococcus faecalis*	2.298
3232	*Enterococcus faecium*	2.403
3233	*Enterococcus faecium*	2.398
3234	*Enterococcus faecium*	2.453
3275	*Enterococcus faecium*	2.258
3276	*Enterococcus faecium*	2.575
3277	*Enterococcus gallinarum*	1.924
3278	*Enterococcus mundtii*	2.282
3280	*Enterococcus faecium*	2.242
3281	*Enterococcus faecalis*	1.954
3282	*Enterococcus faecalis*	2.171
3283	*Enterococcus faecalis*	2.252
3284	*Enterococcus faecium*	2.201
3285	*Enterococcus faecium*	2.069
3286	*Enterococcus faecium*	2.115
3287	*Enterococcus faecium*	2.108
3288	*Enterococcus gallinarum*	2.091
3289	*Enterococcus faecium*	2.238
3290	*Enterococcus hirae*	1.908
3291	*Enterococcus faecium*	2.310
3292	*Enterococcus faecium*	2.149
3293	*Enterococcus faecium*	2.138
3294	*Enterococcus faecium*	2.278
3295	*Enterococcus faecalis*	2.363
3296	*Enterococcus faecium*	2.219
3297	*Enterococcus gallinarum*	2.093
3298	*Enterococcus faecium*	2.255
3299	*Enterococcus casseliflavus*	1.918
3300	*Enterococcus casseliflavus*	2.325
3301	*Enterococcus casseliflavus*	2.126
3302	*Enterococcus faecalis*	2.368
3372	*Staphylococcus sciuri*	1.869
3373	*Enterococcus faecium*	2.395
3374	*Enterococcus faecium*	2.094
3375	*Enterococcus faecium*	2.517

Table 1. MALDI-TOF MS identification and associated scores.

3376	*Enterococcus hirae*	2.027
3377	*Enterococcus hirae*	2.346
3378	*Enterococcus faecium*	2.102
3379	*Enterococcus faecium*	2.341
3380	*Enterococcus faecium*	2.470
3381	*Enterococcus faecium*	2.294
3382	*Enterococcus faecalis*	2.367
3383	*Enterococcus faecium*	2.395
3384	*Enterococcus faecium*	2.369
3385	*Enterococcus faecium*	2.148
3386	*Enterococcus faecium*	2.074
3387	*Enterococcus faecium*	2.452
3388	*Enterococcus faecium*	2.352
3389	*Enterococcus hirae*	2.188
3390	*Enterococcus faecium*	2.289
3391	*Enterococcus hirae*	2.296
3392	*Enterococcus faecium*	2.468
3393	*Enterococcus faecium*	2.470
3394	*Enterococcus hirae*	2.516
3395	*Enterococcus faecium*	2.256
3396	*Enterococcus hirae*	2.400
3475	*Enterococcus hirae*	2.291
3476	*Enterococcus hirae*	2.361
3477	*Enterococcus hirae*	2.266
3478	*Enterococcus hirae*	2.351
3479	*Enterococcus hirae*	2.107
3480	*Enterococcus hirae*	2.436
3481	*Enterococcus hirae*	2.373
3482	*Enterococcus hirae*	2.508
3483	*Enterococcus hirae*	2.415
3484	*Enterococcus hirae*	2.438
3485	*Enterococcus hirae*	2.412
3486	*Enterococcus faecium*	2.175
3487	*Enterococcus hirae*	2.277
3488	NO RELIABLE ID	1.682
3489	*Enterococcus casseliflavus*	1.934
3490	*Enterococcus hirae*	2.235
3492	*Enterococcus hirae*	2.280
3493	*Enterococcus hirae*	2.383
3494	*Enterococcus hirae*	2.370

Table 1. MALDI-TOF MS identification and associated scores.

3495	*Enterococcus faecium*	2.430
3496	*Enterococcus hirae*	2.409
3497	*Enterococcus faecium*	2.311
3498	*Enterococcus gallinarum*	2.206
3499	*Enterococcus hirae*	2.340
3500	*Enterococcus hirae*	2.474
3501	*Enterococcus faecium*	2.153
3502	*Staphylococcus aureus*	2.320
3503	*Enterococcus faecium*	2.310
3504	*Enterococcus hirae*	2.453
3506	*Enterococcus hirae*	2.264
3507	*Enterococcus faecalis*	2.429
3508	*Enterococcus hirae*	2.364
3509	*Enterococcus hirae*	2.419
3510	*Enterococcus hirae*	2.440
3511	*Enterococcus faecium*	2.313
3512	*Enterococcus hirae*	2.379
3513	*Enterococcus casseliflavus*	2.286
3514	*Enterococcus hirae*	2.459
3515	*Enterococcus faecium*	2.520
3516	*Enterococcus faecalis*	2.349
3517	*Enterococcus hirae*	2.461
3518	*Enterococcus faecalis*	2.423
3519	*Enterococcus faecalis*	2.362
3520	*Enterococcus hirae*	2.382
3521	*Enterococcus faecalis*	2.368
3522	*Enterococcus hirae*	2.438
3523	*Enterococcus casseliflavus*	2.321
3524	*Enterococcus hirae*	2.377
3525	*Enterococcus hirae*	2.345
3526	*Enterococcus hirae*	2.465
3527	*Enterococcus hirae*	2.420
3528	*Enterococcus faecalis*	2.422
3529	*Enterococcus faecalis*	2.380
3530	*Staphylococcus aureus*	2.232
3531	*Enterococcus faecium*	2.238
3532	*Enterococcus gallinarum*	2.107
3533	*Enterococcus faecalis*	2.387
3534	*Enterococcus hirae*	2.367
3535	*Enterococcus faecium*	2.236

Table 1. MALDI-TOF MS identification and associated scores.

3536	*Enterococcus hirae*	2.442
3537	*Enterococcus faecium*	2.450
3538	*Enterococcus hirae*	2.299
3540	*Enterococcus hirae*	2.446
3541	*Enterococcus hirae*	2.415
3542	*Enterococcus faecium*	2.205
3543	*Enterococcus hirae*	2.469
3566	*Enterococcus gallinarum*	2.123
3567	*Enterococcus faecium*	2.319
3568	*Enterococcus faecalis*	2.482
3569	*Enterococcus faecium*	2.279
3570	*Enterococcus faecium*	2.331
3571	*Enterococcus faecalis*	2.413
3572	*Enterococcus faecium*	2.388
3573	*Enterococcus hirae*	2.270
3574	*Enterococcus faecalis*	2.393
3575	*Enterococcus hirae*	2.390
3576	*Enterococcus hirae*	2.447
3621	*Enterococcus faecium*	2.220
3622	*Enterococcus hirae*	2.434
3623	*Enterococcus faecium*	2.307
3624	NO RELIABLE ID	1.386
3625	*Enterococcus hirae*	2.452
3626	*Enterococcus faecium*	2.344
3627	*Enterococcus faecium*	2.340
3628	*Enterococcus faecium*	2.274
3629	*Enterococcus faecium*	2.284
3630	*Enterococcus faecalis*	2.443
3631	*Enterococcus faecalis*	2.394
3632	*Enterococcus faecium*	2.293
3633	*Enterococcus faecium*	2.296
3634	*Enterococcus faecalis*	2.419
3635	*Enterococcus faecalis*	2.455
3636	*Enterococcus hirae*	2.443
3637	*Enterococcus hirae*	2.412
3724	*Enterococcus faecalis*	2.384
3725	*Staphylococcus sciuri*	2.076
3726	*Staphylococcus sciuri*	2.201
3727	*Staphylococcus sciuri*	2.293
3728	*Staphylococcus sciuri*	2.373

Table 1. MALDI-TOF MS identification and associated scores.
